# Therapeutic Potential of Vagus Nerve Stimulation for Inflammatory Bowel Diseases

**DOI:** 10.3389/fnins.2021.650971

**Published:** 2021-03-22

**Authors:** Bruno Bonaz, Valérie Sinniger, Sonia Pellissier

**Affiliations:** ^1^Division of Hepato-Gastroenterology, Centre Hospitalier Universitaire Grenoble Alpes, Grenoble, France; ^2^Grenoble Institute of Neurosciences, Inserm U1216, University Grenoble Alpes, Grenoble, France; ^3^Laboratoire Inter-Universitaire de Psychologie Personnalité, Cognition, Changement Social, University Grenoble Alpes, University Savoie Mont Blanc, Grenoble, France

**Keywords:** cholinergic anti-inflammatory pathway, heart rate variability, inflammatory bowel diseases, TNF, vagus nerve, vagus nerve stimulation

## Abstract

The vagus nerve is a mixed nerve, comprising 80% afferent fibers and 20% efferent fibers. It allows a bidirectional communication between the central nervous system and the digestive tract. It has a dual anti-inflammatory properties via activation of the hypothalamic pituitary adrenal axis, by its afferents, but also through a vago-vagal inflammatory reflex involving an afferent (vagal) and an efferent (vagal) arm, called the cholinergic anti-inflammatory pathway. Indeed, the release of acetylcholine at the end of its efferent fibers is able to inhibit the release of tumor necrosis factor (TNF) alpha by macrophages via an interneuron of the enteric nervous system synapsing between the efferent vagal endings and the macrophages and releasing acetylcholine. The vagus nerve also synapses with the splenic sympathetic nerve to inhibit the release of TNF-alpha by splenic macrophages. It can also activate the spinal sympathetic system after central integration of its afferents. This anti-TNF-alpha effect of the vagus nerve can be used in the treatment of chronic inflammatory bowel diseases, represented by Crohn’s disease and ulcerative colitis where this cytokine plays a key role. Bioelectronic medicine, via vagus nerve stimulation, may have an interest in this non-drug therapeutic approach as an alternative to conventional anti-TNF-alpha drugs, which are not devoid of side effects feared by patients.

## Introduction

The vagus nerve, cited as the pneumogastric nerve or 10th cranial nerve, although referred in the singular is paired (right and left VN). It is the longest nerve in the body, extending from the medulla oblongata to the digestive tract. The VN is a mixed nerve containing afferent (sensory) and efferent (motor) nerve fibers. It ensures the innervation of many organs such as the pharynx, larynx, thoracic viscera (heart and lungs) and the digestive tract from the esophagus to the recto-colon. The VN is the main component of the cranial parasympathetic nervous system. The other parasympathetic component is represented by the sacral parasympathetic nucleus (S2–S4) at the origin of the pelvic nerves that provide innervation to the pelvic organs such as the bladder, genitals, and left recto-colon. These two components are part of the ANS (Langley, 1921) comprising the sympathetic and parasympathetic systems, which are classically antagonistic. Due to its mixed character, the VN ensures a bidirectional communication between the CNS and the viscera, in particular the digestive tract in the context of the brain–gut axis (Bonaz et al., 2017a). This reciprocal relationship ensures an integrated and coordinated functioning of digestive functions such as motility, sensitivity, secretion, permeability, immunity. The functioning of the digestive tract is most often unconscious (i.e., not perceived) but can, under certain conditions, become pathological (i.e., perceived as painful). Therapies targeting the VN, whether drugs, nutritional, complementary medicines, or using VN stimulation (VNS), known as Bioelectronic Medicine, could be used in the management of gastrointestinal disorders (Bonaz et al., 2017b). Bioelectronic medicine is based on neuromodulation of the nervous system restoring organ functions and health with less adverse effects than drugs, thus minimizing adherence issues (Olofsson and Tracey, 2017). In particular, due to its anti-inflammatory role, the VN could be used as a non-drug therapy in chronic IBD represented by CD and UC (Bonaz et al., 2016b). Indeed, the VN exerts a dual anti-inflammatory effect: both through its afferents, by stimulating the HPA axis and the release of glucocorticoids from the adrenal glands, and its efferents, through the CAP, more recently described.

## Functional Neuroanatomy of the Vagus Nerve

The VN runs from the brainstem through the neck to many peripheral organs, including the lungs, heart, liver, stomach, intestines. The VN is a mixed nerve consisting of 80% afferent fibers, carrying information from the digestive tract to the CNS, and 20% efferent fibers involved in the control of gastrointestinal functions (Prechtl and Powley, 1990), as well as heart and lungs. Thus, the VN is a major component of the bidirectional communication between the brain and the gut through the brain–gut axis. We will now discuss only the GI functions of the VN.

### Vagal Afferent Fibers

**FIGURE 1:**
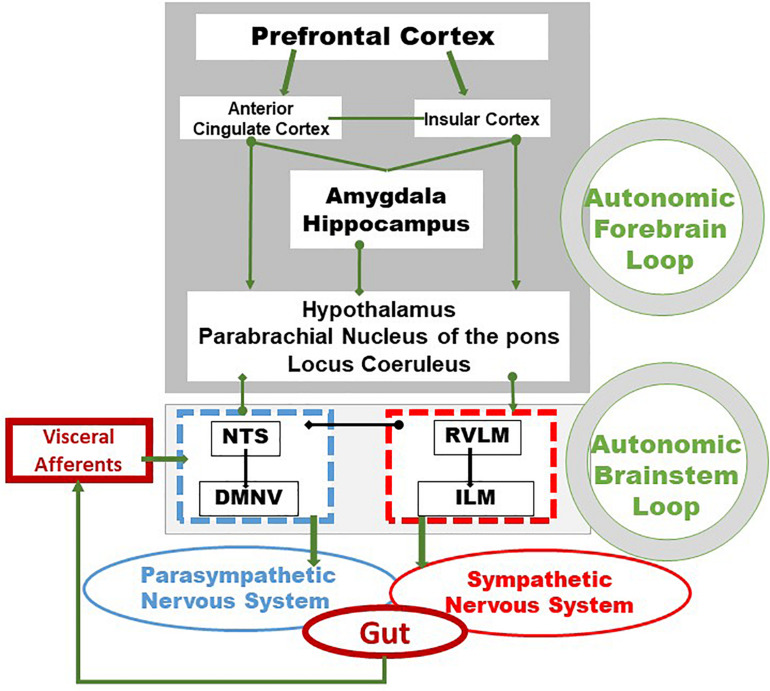
Integrative pathways of the brain–gut axis. Vagal and splanchnic digestive afferents connect to the nucleus tractus solitarius, in close relationship with the dorsal motor nucleus of the vagus, from which vagal efferents originate, thus composing an autonomic loop of the brainstem, involved in the regulation of instinctual motility, acid secretion, food intake and satiety. This loop is modulated by the autonomic loop comprising the hypothalamus, the hippocampus, the amygdala, the anterior cingulate and the insular and prefrontal cortex. This last loop receives, coordinates and integrates visceral information enabling neuroendocrine, emotional, cognitive and behavioral responses. These two central loops explain how stress, sensations and thoughts can influence the functioning of the intestine and vice versa. Adapted from Bonaz et al. (2019). DMNV, dorsal motor nucleus of the vagus nerve; ILM, intermediolateralis nucleus; NTS, nucleus tractus solitarius; RVLM, rostral ventrolateral medulla.

Vagal afferents inform the CNS, usually unconsciously, of the functional state of the gastrointestinal tract. These afferents originate from free endings in the different layers of the gut wall, including in the external muscle layers, myenteric plexus, and mucosal lamina propria and travel through the VN to the nucleus tractus solitarius (NTS) according to a viscerotopic distribution (Powley et al., 2019). The NTS, the main entry point of the digestive tract into the brain, is located in the medulla, just above the DMNV which is at the origin of vagal efferent fibers with the nucleus ambiguus (Jean, 1991). Thus, the NTS and the DMNV are closely connected. In fact, the dendrites of vagal motor neurons are connected with vagal afferents ending in the NTS, at the origin of vago-vagal reflex loops (Figure 1) (Travagli et al., 2006; Bonaz et al., 2019). Vagal afferent cell bodies are located in the nodose ganglia or jugular ganglia, at the base of the skull. Peripheral stimuli, via vagal afferents ending in the NTS, are transmitted to many regions of the CNS through projections of the NTS onto structures such as the parabrachial nucleus, an important sensory relay of the NTS, the hypothalamus, in particular the PVH, the limbic system including the amygdala, thalamus, hippocampus, and cerebral cortex including the insula and the prefrontal cortex (Norgren, 1978; Sawchenko, 1983; Cechetto, 1987). These different structures are part of the CAN described by Benarroch (1993). The CAN is at the origin of autonomic, behavioral, cognitive, and endocrine responses. It is capable of modulating the functioning of the ANS via descending pathways projecting onto sympathetic pre-ganglionic neurons in the spinal cord and onto the DMNV at the origin of vagal efferents. Vagal afferents are involved in detecting the presence of nutrients and their chemical composition in the digestive tract in the post-prandial period. They contain chemoreceptors, thermoreceptors, osmoreceptors, mechanoreceptors as opposed to afferent spinal fibers which essentially vehiculates pathways of visceral pain of digestive origin to the spinal cord (Berthoud and Neuhuber, 2000). Most of the nervous information coming from the viscera is not conscious but can become so in pathological conditions, particularly inflammatory. The VN is a major component of the pathways of interoception which is the sense of the body’s internal physiological state (Craig, 2002), and interoceptive abnormalities are implicated in the pathophysiology of psychiatric disorders, neurodegenerative and neurological disorders, as well as in somatic disorders of brain-body interactions, including functional digestive disorders and IBD (Fournier et al., 2020; Bonaz et al., 2021).

### Vagal Efferent Fibers

These fibers originate at the level of the medulla oblongata, from pre-ganglionic neurons located in the DMNV and travel through the VN toward the viscera synapsing with a second post-ganglionic neuron located in the target organ, namely the digestive wall. In the digestive tract, this second order neuron is an integral part of the enteric (or intrinsic) nervous system, a real “second brain” or “gut brain,” able to ensure motor and secretory autonomy of the digestive tract (Furness et al., 2014). It is classically stated that the VN innervates the entire digestive tract up to the splenic angle (Netter, 1989). However, for others, it innervates the entire digestive tract in humans (Delmas and Laux, 1933) as well as in rats (Altschuler et al., 1993). The pelvic nerves classically innervate the left colon and the rectum as well as the bladder and genital organs. The sacral (S2–S4) parasympathetic nucleus is under the control of the Barrington’s nucleus, also called the pontine micturition center (Valentino et al., 1999), which lies adjacent to, and interacts with the LC, the brain’s principle noradrenergic projection center, involved in arousal, stress, autonomic function, cognition, and behavior (Benarroch, 2018). The neurotransmitter of the vagal and pelvic parasympathetic system is ACh for pre- and post-ganglionic neurons acting on nicotinic receptors at the pre-ganglionic level and muscarinic receptors at the post-ganglionic level.

Parasympathetic innervation of the gut is involved in the neuroimmune regulation of intestinal barrier through the recruitment of α7 nicotinic ACh receptor (α7nAChRs). It acts on enteroglial cells, interacting with innate immune cells (Fornai et al., 2018), myenteric neurons, making synaptic contacts with resident macrophage expressing α7nAChR. Vagal afferent fibers penetrate to the tips of jejunal villi and some of these nerves make intimate contact with intestinal mucosal mast cells. These data provide the microanatomical basis for direct neural communication between the CNS and mast cells in the gastrointestinal mucosa (Williams et al., 1997). Overall, the stimulation of vagal efferents could obviously “activate” these different gut cells and be one of the component of the CAP.

## Inflammatory Bowel Diseases

Inflammatory bowel diseases are organic diseases classically divided in CD and UC. CD can involve all the digestive tract, from the mouth to the anus, while UC involves the recto-colon only. IBD start early in life (between 15 and 30 years) and evolve by periods of flares alternating with periods of remission of variable duration (Chang, 2020). Symptoms are represented by abdominal pain, diarrhea, fever, weight loss, and extra-intestinal (skin, eyes, joints) manifestations. About 1.5 million Americans and 2.2 million people in Europe are affected by IBD. There is a rising incidence of IBD in Western countries, supporting the hypothesis that “Westernization” of our lifestyle has led to the increased incidence and prevalence of IBD (Molodecky et al., 2012).

The pathophysiology of IBD is multifactorial involving genetic, immunologic, infectious and environmental factors (Chang, 2020). The theory is that genetically susceptible individuals experience an environmental trigger(s), resulting in an inappropriate immune response, potentially against gut microbes. Stress, through brain–gut interactions, as well as environmental factors, based on experimental and clinical data (Bonaz and Bernstein, 2013), has been proposed as a contributor. An imbalance of the ANS is observed in IBD, represented by a sympathetic dysfunction in CD (Lindgren et al., 1991) and a vagal dysfunction in UC (Lindgren et al., 1993). We have recently reported that this dysautonomia may be dependent on psychological adjustment in IBD. Indeed, the equilibrium of the ANS is differentially adapted according to the disease. This equilibrium is conjugated with positive affective and cognitive adjustment in IBD (Pellissier et al., 2010). Presently, there is no treatment to cure IBD. Current treatments suppress disease activity and there is generally a relapse of the disease after discontinuation of the treatment. TNF is a key cytokine that is involved in IBD and anti-TNF therapies have transformed the management of IBD (Peyrin-Biroulet, 2010). New compounds targeting other pro-inflammatory cytokines, such as IL-12, IL-23, anti-integrin therapies, and anti-Janus kinase (JAK) are available (Pagnini et al., 2019; D’Amico et al., 2020). Surgery is an alternative to a failure of treatment or a complication of IBD (perforation, abscess, stenosis) but the disease recurs after surgery. Anti-TNF therapies used in IBD have been shown to be effective but there is a 20–30% primary non-response rate (Ford et al., 2011) and the annual risk of loss of response to anti-TNF is 13% per patient-year for Infliximab (Gisbert and Panes, 2009) and 20% per patient-year for Adalimumab (Billioud et al., 2011). This secondary non-response is due to (i) the development of autoantibodies, in particular for Infliximab which is a chimeric molecule (75% human and 25% mouse), but also for Adalimumab (100% human) to a lesser degree, or (ii) a secondary failure of a well-dosed or under-dosed treatment using therapeutic drug monitoring (Ben-Horin and Chowers, 2011; Chaparro et al., 2012). Anti-TNF treatment currently represents the bulk of the cost of IBD treatment (van der Valk et al., 2014). Indeed, the median cost of a 1-year anti-TNF therapy raises up to $40,000 for CD patients (Targownik et al., 2019). In addition, biological therapies are not devoid of numerous side effects with a major impact on the patient’s quality of life (Kerbleski and Gottlieb, 2009; Pereira et al., 2015; Murdaca et al., 2016). As a result, by fear of these side effects and the need for chronic treatment of these pathologies, patients are increasingly reluctant to take these treatments and to continue them once they are in remission. This explains, in particular, the 30–50% of non-adherence (Chan et al., 2017) and the growing interest of the patients for complementary medicines (Torres et al., 2019).

**FIGURE 2:**
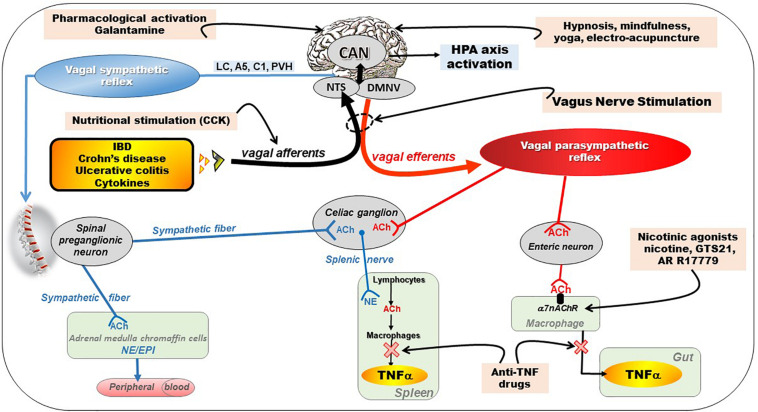
Different pathways of the anti-inflammatory properties of the vagus nerve: (1) through activation of the HPA axis via vagal afferents and through vago-parasympathetic efferents (red), (2) through sympathetic efferents (blue) arising from thoraco-lumbar spinal preganglionic neurons through the vago-sympathetic pathway where vagal afferents activate central descending pathways (e.g., LC, A5, C1, PVH) targeting spinal pre-ganglionic neurons. Targeting the VN for its anti-inflammatory properties (pink) in chronic inflammatory diseases (orange) such as inflammatory bowel diseases appears as potentially effective therapeutics. Adapted from Bonaz et al. (2017a). A5, A5 noradrenergic group (in the brainstem); ACh, acetylcholine; C1, C1 adrenergic group (in the brainstem); CAN, central autonomic network; CCK, cholecystokinin; DMNV, dorsal motor nucleus of the vagus nerve; EPI, epinephrine; HPA, hypothalamic–pituitary–adrenal; IBD, inflammatory bowel diseases; LC, locus coeruleus; NE, norepinephrine; NTS, nucleus tractus solitarius; PVH, paraventricular nucleus of the hypothalamus; TNFα, tumor necrosis factor-alpha; α7nAChR, alpha7nicotinic acetylcholine receptor.

Consequently, a treatment targeting pro-inflammatory cytokines such as TNF-alpha and others, exploiting the CAP, with few side effects, devoid of problem of compliance, and cheaper than biologicals (i.e., anti-TNF-alpha) would be of great value. In this context, targeting the anti-inflammatory properties of the VN would be of interest (Figure 2). In particular, VNS, as a non-drug therapy could serve as an alternative to classical biological therapies. We have shown recently that there is a specific homeostatic link between vagal tone and TNF-alpha in CD patients since a low vagal tone was associated with a high level of TNF in the plasma (Pellissier et al., 2014). In addition, since stress is classically known to stimulate the sympathetic nervous system, which has a pro-inflammatory effect, and to inhibit the VN (Wood and Woods, 2007), and thus the CAP, VNS may help to restore an equilibrium of the sympatho-vagal balance.

## Anti-Inflammatory Properties of the Vagus Nerve

The VN has a double anti-inflammatory effect both via its afferents and efferents (Bonaz and Bernstein, 2013; Bonaz et al., 2017a).

### Anti-inflammatory Properties of Vagal Afferents

The VN is a key element of the neuro-endocrine immune axis whose purpose is to ensure a homeostasis balance. The peripheral release of pro-inflammatory cytokines such as IL-1beta, IL-6, and TNF, activates vagal afferents via their interaction with receptors on the para-nodes (Goehler et al., 1997) (Figure 2). Zanos et al. (2018) have very recently shown that electrical signals recorded on the cervical VN can be decoded to discriminate between cytokine-specific signals. In animals, the induction of a septic shock by systemic or intraperitoneal injection of LPS, components of the wall of Gram-negative bacteria, leads to the release of these cytokines by blood and/or tissue macrophages, which in turn activates vagal afferents leading to fever, sleep and aphagia (Dantzer et al., 1998). This signal activates the HPA axis after being integrated at the level of the NTS and then transmitted to the hypothalamus via projections to the PVH, more particularly on CRF neurons, the main neuro-mediator of stress, located in the parvocellular zone of the PVH (Rivest et al., 2000) (Figure 2). These CRF neurons project themselves into the pituitary gland, whose activation will release ACTH that will stimulate the release of glucocorticoids (cortisol) by the adrenal glands with well-known anti-inflammatory properties. Most of neuromodulation studies focus on vagal regulation of inflammation via the peripheral efferent pathway toward the viscera. However, abdominal vagal afferent neurostimulation suppresses systemic inflammation in rats, and the efferent neural pathway for this action is in the splanchnic sympathetic nerves (Komegae et al., 2018). Vagal stimulation also modulates arthritic joint inflammation through an afferent pathway mediated by the LC where central stimulation is followed by activation of joint sympathetic nerve terminals releasing NE. The vagal control of arthritic joint inflammation is dampened by selective adrenergic beta-blockers. These results reveals a novel neuro-immune brain map with afferent vagal signals controlling side-specific articular inflammation through specific inflammatory-processing brain centers and joint sympathetic innervations (Bassi et al., 2017).

### Anti-inflammatory Properties of Vagal Efferents

In 2000, Kevin Tracey’s team described, for the first time, the CAP (Borovikova et al., 2000; Pavlov et al., 2003). This group showed that a septic shock in rats, induced by iv injection of LPS, was prevented by low frequency neurostimulation of the distal end cut of the VN thus stimulating vagal efferents (Borovikova et al., 2000). The authors concluded that there was an inflammatory reflex in which stimulation of vagal afferents by pro-inflammatory cytokines resulted in activation of vagal efferents that inhibited the release of these cytokines by tissue macrophages, in particular TNF but also other proinflammatory cytokines such as IL6, IL1β but not the anti-inflammatory cytokine IL-10. The VN has therefore anti-inflammatory properties through the inhibition of pro-inflammatory cytokines (Figure 2). This group also characterized the cholinergic receptor of macrophages involved in this effect, which was not muscarinic but α7nAChR. Indeed, the effect of VNS was abolished in animals knock out for this receptor (Wang et al., 2003). Intracellular mechanisms downstream of α7nAChR activate the JAK2–signal transducer and activator of transcription 3 pathway, sequester Nuclear Factor-kB (NF-κB), and inhibit activation of the inflammasome (Guarini et al., 2003; de Jonge et al., 2005; Lu et al., 2014). This anti-TNF effect of the VN has, of course, therapeutic applications in pathologies where this cytokine is involved such as IBD. However, this effect of the VN is not direct via vagal efferent endings on macrophages but indirect through the interaction of the VN with nNOS, VIP and ChAT enteric neurons located within the gut muscularis with nerve endings in close proximity of the resident macrophages (Cailotto et al., 2014). Tracey’s group also showed that the spleen, an important source of ACh, where it was first isolated, and TNF in the body, was also involved in the anti-inflammatory effect of the VN. This effect involves a connection between vagal efferent endings and the spleen (Rosas-Ballina et al., 2008), through the celiac sympathetic ganglion, inducing the release of NE by noradrenergic endings in the spleen (Figure 2). They recently showed that cholinergic neurons in the DMNV, which project to the celiac superior mesenteric ganglia, significantly increase splenic nerve activity and inhibit TNF production (Kressel et al., 2020). So there is a vago-sympathetic excitatory pathway while the VN and the sympathetic nervous system have generally antagonistic effects. Indeed, NE acts on beta2 receptors of splenic lymphocytes to release ACh by these lymphocytes: this is the non-neuronal cholinergic pathway by opposition to the neuronal (i.e., vagal) cholinergic pathway (Figure 2). ACh then binds to α7nAChR of splenic macrophages to inhibit the release of TNF by these macrophages (Olofsson et al., 2012). These activated T lymphocytes do not just inhibit macrophages locally. They may also move and behave like “mobile neurons” and downregulate inflammation in areas not innervated by the VN. The characteristics of these CHAT-containing T cells have been well outlined by the Tracey’s group (Rosas-Ballina et al., 2011; Olofsson et al., 2016). However, the innervation of the spleen by the VN via this interaction with the splenic nerve is questioned by some authors, notably by the work of Martelli et al. (2014). For this author, the efferent anti-inflammatory pathway is not the VN but the sympathetic system, notably the splanchnic sympathetic nerve and its anti-inflammatory effect is distributed across abdominal organs (Martelli et al., 2019). The sympathetic nervous system originates in the thoracolumbar spinal pre-ganglionic neurons (T1-L2) and synapses with post-ganglionic neurons at the origin of splanchnic sympathetic nerves distributed to the viscera, with a mirror distribution of the parasympathetic system (Figure 2). These pre-ganglionic spinal sympathetic neurons are under the control of central nuclei such as the PVH, Kõlliker-Fuse nucleus, pontine noradrenergic groups A5, the LC (A6), the chemosensitive region of the ventral medulla, and possibly the region of the A1 catecholamine cell group (Loewy, 1981). These neural groups are part of the CAN and send descending projections to modulate these pre-ganglionic sympathetic neurons. In particular, Abe et al. (2017) showed, in an experimental model of renal inflammation, that C1 was involved in an anti-inflammatory reflex involving only the splanchnic nerve.

In fact, contrasting the vagal anti-inflammatory pathway with the splanchnic pathway is a reductive view since both pathways can act in concert to play a pivotal role in the crosstalk with the immune system, a fortiori if they are activated by VNS (Brinkman et al., 2019). In particular, because of its mixed, essentially afferent, character, the VN via its central projections on the NTS and then on the CAN is able to activate A5, A6, and C1 which will secondarily activate the splanchnic sympathetic nerves via their descending projections to the spinal cord. Our group has shown in particular that low-frequency (5Hz) VNS in rats, supposed to activate only vagal efferents, also activated vagal afferents (Reyt et al., 2010). Lomarev et al. (2002) have also shown that, compared to 5 Hz, 20 Hz VNS produced more acute activity changes from rest in regions similar to our initial VNS synchronized fMRI feasibility study in depression. There is therefore a bidirectional activation of vagal fibers by VNS, even at low frequency, involving a peripheral vagal and sympathetic action via a vago-vagal and vago-sympathetic reflex through the CNS (Bonaz et al., 2019).

## The Vagus Nerve at the Interface of the Microbiota–Gut–Brain Axis

**FIGURE 3:**
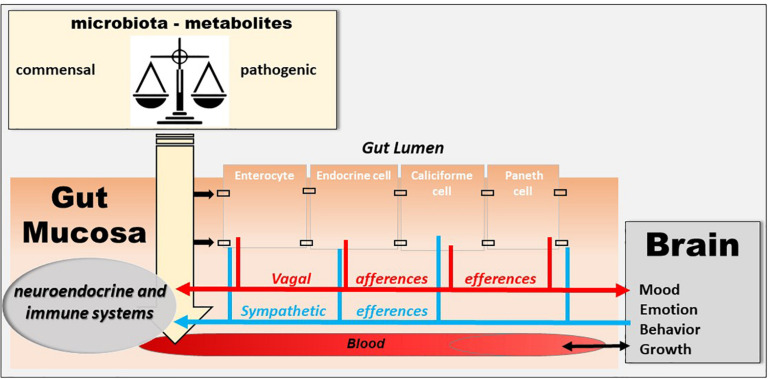
The microbiota–gut–brain axis. The microbiota exerts an effect on the gut-brain axis, impacting the biochemistry of the peripheral and central nervous system. Commensal and/or pathogenic bacteria and their metabolites are translocated across the intestinal barrier and act on both the digestive immune system and vagal afferents. Similarly, the brain acts on the various organs, including the digestive tract, and can thus regulate the survival and proliferation of the intestinal microbiota.

The human intestine contains 10^13^ to 10^14^ microorganisms, which is much more than the cells in our body and 100 times more genes than our genome. The weight of the microbiota is about 1 kg in adults, approximately the weight of the human brain. In healthy subjects, two species of bacteria, Bacteroides and Firmicutes, dominate the bacterial composition, with smaller amounts of actinobacteria, proteobacteria and verrucomicrobia. At the species level, each individual presents a very specific signature. In addition to bacteria, the intestinal microbiota contains methanogenic archaea, eukaryotes (mainly yeasts) and numerous phages (Eckburg et al., 2005; Reyes et al., 2010). Recently, it has been shown that the microbiota, the gut, and the brain communicate via the microbiota–gut–brain axis (Cryan et al., 2019) and that a disruption of this axis is involved in the pathophysiology of neurodegenerative diseases (Cryan and Dinan, 2012; Forsythe et al., 2016). A dysbiosis is observed in various pathological conditions of the gastrointestinal tract, such as IBD (Sundin et al., 2017), and fecal transplantation is presently under investigation in clinical trials (Levy and Allegretti, 2019). However, the question is whether it is a cause or consequence of an abnormal communication between the gut and the brain. The VN is a key component of this microbiota–gut–brain axis (Bonaz et al., 2018) (Figure 3). Indeed, it is able to detect metabolites of the microbiota through its afferents and to transfer this intestinal information to the CAN, then generating an adapted or inappropriate response from the CAN to the intestine and the microbiota (Bonaz et al., 2018). It can also be activated indirectly by the microbiota via the interaction of the microbiota with digestive endocrine cells that will release serotonin acting on 5-HT3 receptors of vagal afferents. The VN, via the CAP, could modulate the intestinal microbiota by decreasing intestinal permeability and modulating local immunity (Bonaz et al., 2018). One can imagine that, an indicator such as HRV, easy to assess through the detection of heartbeat intervals, could be used to explore the microbiota–gut–brain axis homeostasis. Indeed, HRV is probably not a direct index of “the true” vagal tone (Marmerstein et al., 2021) since it is an indirect assessment of the parasympathetic modulation on the heart so that the metrics of the HRV would rather reflect different aspects of the neurophysiologic regulation of the heart rhythm (Reyes del Paso et al., 2013; Thomas et al., 2019). Presently, HRV is the final output of several regulatory loops resulting from afferent signals integrated at the level of the CNS influencing the efferent vagally mediated modulations on the heart (Thayer and Lane, 2009). In that way, HRV can be used to mark and follow the activity of the neurophysiological pathways involved in adaptation, homeostasis and health (Thayer et al., 2012). A recent meta-analysis underlines that HRV metrics such as the high frequency band (HF-HRV) and the standard deviation of RR intervals (SDNN) are the most robustly associated with inflammation (Williams et al., 2019). Finally, targeting the VN through VNS, even if the mechanism is not clear yet, could have therapeutic implications in the modulation of the microbiota–gut–brain axis.

## Stress and the Vagus Nerve

**FIGURE 4:**
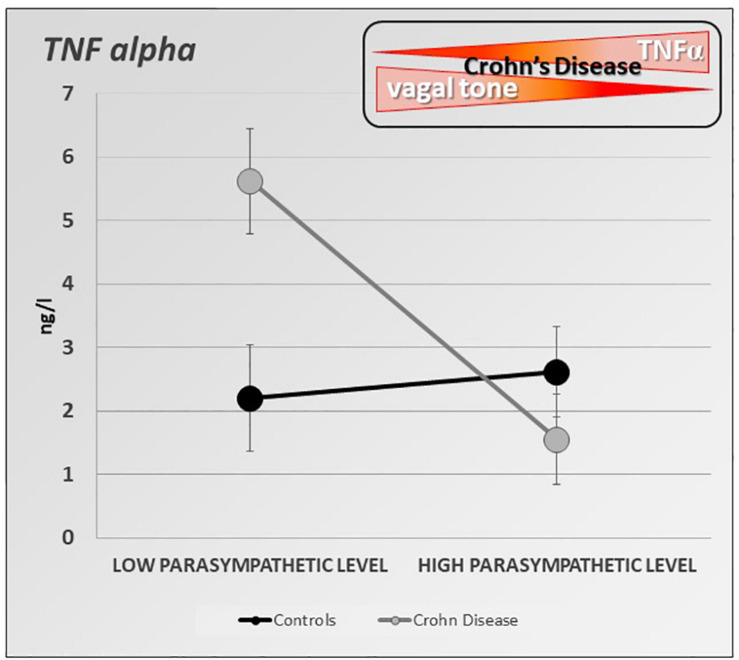
Specific inverse relationship between the resting parasympathetic vagal tone and TNF-alpha plasma level in Crohn’s disease (CD) patients. CD patients with high parasympathetic vagal tone exhibit a lower level of TNF-alpha than those with low parasympathetic vagal tone. Parasympathetic vagal tone was assessed by power spectral analysis of HRV and TNF-alpha level was assessed by ELISA-based technic. Data expressed as mean ± sem (adapted from Pellissier et al., 2014).

Besides its well-known effects on gastrointestinal motility (Tache et al., 2001), stress increases intestinal permeability, modifies the intestinal flora, and intestinal immunity (Tache and Bonaz, 2007; Larauche et al.,2009a,b). If the role of stress is well known in functional digestive disorders such as irritable bowel syndrome (Pellissier and Bonaz, 2017), recent data have shown its role in IBD (Bonaz and Bernstein, 2013). Furthermore, stress modifies the activity of the ANS. Classically, stress leads to vagal inhibition and activation of the sympathetic system, thus favoring the body’s ability to cope with a “threat to its homeostasis” and in fact strengthens the defenses through the pro-inflammatory reaction (Tache and Bonaz, 2007). Any vagal hypotonia with or without sympathetic hypertonia can therefore promote an inflammatory process. In patients at the pre-rheumatoid arthritis stage, those who had vagal hypotonia developed rheumatoid arthritis more easily afterward (Roura et al., 2016). Moreover, several arguments are in favor with such a causal association in IBD. First, a low level of HRV as indexed by the RMSSD metric, at onset of newly diagnosed UC patients predicts the systemic inflammatory response over 3 years of follow-up (Gunterberg et al., 2016). Second, there is a positive association between vagotomy and later IBD, and this particularly for CD revealing the role of VN integrity in the prevention of IBD (Liu et al., 2020). Thus, we can assume that the level of vagal tone is predictive of the development of an inflammatory disorder in people at risk. We also showed in patients with CD in remission, that a low resting vagal tone correlated with elevated circulating TNF (Pellissier et al., 2014) (Figure 4). The systemic inflammation observed during IBD or other chronic inflammatory pathologies is capable of leading to vagal hypotonia, which in turn maintains this inflammatory state. Moreover, chronic inflammation, by its central impact, can lead to a depressive state which itself can promote an inflammatory flare-up of the disease (Mikocka-Walus et al.,2016a,b; Bullmore, 2018). Two meta-analysis have shown that the levels of proinflammatory cytokines, such as TNF, IL-6, IL-1, and CRP, increase during depressive episodes (Hiles et al., 2012; Kohler et al., 2017). Chronic infection or stress may inhibit the negative feedback of the HPA axis, triggers the activation of microglia in the brain, and increases the permeability of the blood–brain barrier, resulting in excessive activation of proinflammatory cytokines (Song and Wang, 2011). Increased proinflammatory cytokines may cause MDD by activating the HPA axis, which results in a depletion of serotonin with an increased activity of the indoleamine-2,3-dioxygenase enzyme in the tryptophan–kynurenine system (Schiepers et al., 2005). Anti-inflammatory agents improve depressive symptoms compared to placebo as add-on in MDD patients and as monotherapy (Köhler-Forsberg et al., 2019). There is a link between depression and inflammation as a vicious circle: depression promotes inflammation and vice-versa. Depression increases the risk of IBD, which may be mitigated by the use of anti-depressants in the treatment of depression (Frolkis et al., 2019). Recently, it has been shown that individuals with IBD have a higher prevalence of depression than matched controls as early as 9 years before diagnosis (Blackwell et al., 2020). Depression in the absence of prior gastrointestinal symptoms was not associated with a future diagnosis of IBD but those patients with depression diagnosed after already experiencing gastrointestinal symptoms are at increased risk of later being diagnosed with IBD. The excess prevalence of depression prior to a diagnosis of IBD may be a consequence of diagnostic delay and untreated gastrointestinal symptoms. Antidepressant drugs have also shown a definite interest in the management of IBD (Macer et al., 2017). Therefore, any therapy, whether drug-based or not such as VNS, capable of restoring vagal activity, has a potential interest in IBD, but also in other chronic non-digestive inflammatory pathologies such as depression. There is a reduced HRV in depression with a high risk of cardiovascular disease (Sgoifo et al., 2015). Recently, it has been shown that transcranial direct current stimulation as an adjunct therapy is effective in alleviating unipolar and bipolar depression. Moreover, the amplitude of the increase in parasympathetic signaling, as indexed by the RR interval lengthening, during the first session, predicts the clinical response after 10-sessions (Lin et al., 2021).

## How to Use the Anti-Inflammatory Properties of the Vagus Nerve for Therapeutic Purposes?

Targeting the VN for anti-inflammatory purposes can be done in different ways (Bonaz and Bernstein, 2013; Bonaz et al., 2017a) (Figure 2).

### Pharmacological Stimulation of Alpha7 Nicotinic Receptors

Pharmacologic stimulation can be performed with α7nAChR agonists such as GTS-21, AR-R17779 which have been used in post-operative ileus models, following intestinal macrophagic activation at the origin of ileus, as well as in experimental pancreatitis (van Westerloo et al., 2006; The et al., 2007). GTS-21, is able to inhibit proinflammatory cytokines *in vitro* and *in vivo* and improve survival in murine endotoxemia and severe sepsis (Pavlov et al., 2007). Hyperoxia-induced acute lung injury is attenuated by GTS-21 by inhibiting extracellular high mobility group box 1-mediated inflammatory responses (Sitapara et al., 2020).

### Nutritional Stimulation

Cholecystokinin, a satiety hormone released at the duodenal level by I cells by the arrival of fat during the meal, acts on vagal afferents receptors. Activation of these receptors by cholecystokinin stimulates vagal afferents and an inflammatory reflex via activation of the CAP. This has been demonstrated in a model of hemorrhagic shock resulting in systemic release of proinflammatory cytokines such as TNF and IL-6 and inducing intestinal permeability through a loss of intestinal barrier function. The ingestion of high-fat enteral nutrition inhibited the release of these cytokines and reduced intestinal permeability. This effect was prevented either by vagotomy or by antagonists targeting cholecystokinin receptors or α7nAChR (Luyer et al., 2005).

### Fasting

Fasting has a well-known anti-inflammatory effect, especially in IBD. This effect could be conveyed by ghrelin, an orexigenic peptide released during fasting by P/D1 cells of the gastric fundus, considered to be a leptin antagonist and also known for its gastric pro-kinetic properties (Mao et al., 2015). Plasma ghrelin levels increase during fasting and decrease after ingesting glucose and lipid, but not protein. The efferent VN contributes to the fasting-induced increase in ghrelin secretion. Ghrelin secreted by the stomach stimulates vagal afferents and promotes food intake (Nonogaki, 2008). As an illustration, mice invalidated for ghrelin have a suppression of the CAP demonstrated by a reduction in VN activity and an increase in plasma levels of the pro-inflammatory cytokines IL-1beta and IL-6. This effect is prevented by the administration of ghrelin or nicotine, which activate this anti-inflammatory pathway (Mao et al., 2015). Ghrelin stimulates the VN since vagotomy dampens its effects (Zhou et al., 2020). Ghrelin protects animals from renal ischemia-reperfusion injury through the VN (Rajan et al., 2012).

### Stimulation of Central Cholinergic Pathways

Galantamine, a selective acetylcholinesterase inhibitor which has shown potential for the treatment of Alzheimer’s disease, is able to cross the blood–brain barrier, after peripheral administration (Metz and Pavlov, 2020). Galantamine stimulates the central cholinergic system, activates the VN and inhibits the peripheral release of TNF during endotoxic shock in animals. This effect is prevented by a centrally acting muscarinic antagonist, or in α7nAChR knockout mice (Pavlov et al., 2009). These findings show that inhibition of brain acetylcholinesterase suppresses systemic inflammation through a central muscarinic receptor-mediated and vagal- and α7nAChR-dependent mechanism. Galantamine also improves experimental colitis in mice; this effect is suppressed by vagotomy, splenectomy or splenic denervation (Ji et al., 2014). Using forebrain-selective genetic ablation of ACh release and vagotomy, selective stimulation of ACh action on the M1 muscarinic ACh receptor (M1 mAChR), as well as selective optogenetic stimulation of basal forebrain cholinergic neurons innervating brain regions with abundant M1 mAChR localization, Lehner et al. (2019) have shown that forebrain cholinergic neurons regulate innate immune responses and inflammation. Thus, targeting forebrain cholinergic signaling should be of interest in cholinergic dysfunction diseases.

### Physical Activity

Exercise reduces systemic inflammatory activity (Heffernan et al., 2009). Regular physical activity reduces the risk of developing chronic diseases involving inflammation. Its mechanism of action is not well known but it may involve the CAP, as high levels of physical activity are associated with increased vagal tone and low levels of CRP, a systemic inflammatory marker (Lujan and DiCarlo, 2013). Physical activity therefore has a potential anti-inflammatory effect in inflammatory pathologies whether used in isolation or in combination with treatment. Physical activity, by enhancing parasympathetic tone and activating the CAP, is a therapeutic strategy to restrain chronic inflammation and prevent many chronic diseases (Lujan and DiCarlo, 2013). The anti-inflammatory effect of physical activity has also been associated with an enhancement of the sympathetic output (Weippert et al., 2013).

### Complementary Techniques

*Hypnosis* stimulates the VN as shown in the study of HRV under hypnosis; HRV decreased during hypnosis but increased post-hypnosis (Yuksel et al., 2013). The efficacy of hypnosis is well known in patients with irritable bowel syndrome and some data are available in IBD, where its use is believed to improve patients with UC (Mawdsley et al., 2008) and prolong clinical remission (Keefer et al., 2013). *Mindfulness meditation (“mindfulness”)* is able of activating the VN and may have anti-inflammatory as well as anti-stress properties by increasing HRV (Azam et al., 2015; Lumma et al., 2015). There are possible effects of mindfulness meditation on specific markers of inflammation, cell-mediated immunity, and biological aging, but these results are tentative and require further replication (Black and Slavich, 2016). Regular practice of *yoga* also increases vagal tone (Tyagi and Cohen, 2016). In a model of chronic obstructive pulmonary disease, *electro-acupuncture* can reduce the lung inflammatory response and improve lung function in this model, which may be related to its involvement in the regulation of the CAP (Zhang et al., 2018).

### Vagus Nerve Stimulation for Anti-inflammatory Purposes in Chronic Inflammatory Bowel Diseases

Vagus nerve stimulation is a new therapeutic pathway for TNF-mediated chronic inflammatory diseases (Bonaz et al.,2016a,b) within the framework of Bioelectronic Medicine, whose goal is to use miniaturized stimulators to administer electrical nerve signals for non-drug therapeutic purposes (Olofsson and Tracey, 2017). VNS is already approved for the treatment of drug-refractory epilepsy and depression (Bonaz et al., 2013).

#### Experimental Data

The first data on the anti-inflammatory effect of the VN during digestive inflammation was reported by Miceli and Jacobson (2003). They showed that prior administration of anticholinesterase drugs such as neostigmine, which does not cross the blood–brain barrier, or physostigmine, which does, improved an experimental 2,4,6-trinitrobenzene sulfonic acid (TNBS)-colitis in rats in a model mimicking CD. This effect was more convincing for physostigmine, thus in favor of a predominant central mechanism. Vagotomy aggravated experimental colitis in mice, which is in favor of the protective role of the VN (Ghia et al., 2007). However, intestine-specific vagal nerve denervation had no effect in DSS-induced colitis (Willemze et al., 2018). Our group showed, for the first time, in the non-vagotomized vigilant rat, that low frequency (5 Hz) VNS, 3 h per day for five consecutive days, resulted in an improvement of TNBS-colitis in rats (Meregnani et al., 2011). VNS reduced the degree of body weight loss and inflammatory markers as observed above the lesion by histological score and myeloperoxidase quantification. This anti-inflammatory effect was also demonstrated by the improvement of a multivariate index of colitis (including body weight, temperature and locomotor activity, macroscopic area of lesions, histological, and biological parameters such as myeloperoxidase activity, cytokine and cytokine-related mRNAs). We have also shown that low-frequency stimulation (5 Hz), supposed to stimulate vagal efferents, also stimulated vagal afferents, as demonstrated in a brain imaging (MRI) study in rats where low-frequency neurostimulation led to deactivation in the NTS, the gateway to vagal afferents, and its projection sites (Reyt et al., 2010). Low-frequency VNS therefore stimulates both vagal efferents and afferents, which implies that it stimulates the two anti-inflammatory pathways of the VN: the CAP and the HPA axis, and also probably the anti-inflammatory sympathetic pathway (Martelli et al., 2016; Komegae et al., 2018). Our data were confirmed later by the study of Sun et al. (2013) who also performed chronic VNS *in vivo* in rats with TNBS-colitis, as well as LPS-induced inflammatory response in human epithelial colorectal adenocarcinoma cells (Caco-2) by ACh *in vitro*. They showed that clinical activity index, histological scores, biological inflammation using myeloperoxydase activity, iNOS, TNF and IL-6 levels were significantly decreased by chronic VNS and that vagal activity, measured by HRV, was improved. In addition, both VNS and ACh reduced the phosphorylation of MAPKs and prevented the nuclear translocation of NF-κB p65. Jin et al. (2017) also found an improvement of TNBS-colitis in rats both at the clinical, biological (MPOA, TNF, IL1-beta, IL-6) and histological level. In addition, they showed that the addition of electroacupuncture to VNS may enhanced the anti-inflammatory effect of VNS. Both VNS plus electroacupuncture and VNS alone substantially increased vagal activity, measured by HRV, and decreased sympathetic activity compared with sham (*P* < 0.001, *P* < 0.001, respectively). More recently, Meroni et al. (2018) performed VNS in mice following intracolonic oxazolone administration. This model represents a model of sepsis and may resemble a severe type of UC, resulting in a severe destruction of the colonic mucosa and a rapid drop in body temperature leading to a 65% mortality rate at day 5. Severe infiltration of neutrophils and monocytes was detected 6h after oxazolone administration which was associated with a Th2-type inflammatory response. VNS significantly improved survival rate which correlated with decreased levels of HMGB1 and reduced colonic (IL-6 and CXCL1 mRNA) and serum cytokine levels (IL-6, TNF, and CXCL1) compared to sham treated mice. Chen et al. (2018) performed a dextran sodium sulfate (DSS) colitis in mice mimicking UC. They showed that VNS improved DSS-colitis but also alleviated cerebral cortical microinfarct induced by a two-photon laser and this neuroprotection was associated with the suppression of blood–brain barrier permeability, neuroinflammation and oxidative stress.

#### Clinical Data

In a translational approach from bench to bedside, we conducted, for the first time, a pilot study of VNS in patients with moderate to severe CD as an alternative to anti-TNF drug therapy or in treatment-naïve patients (ClinicalTrials.gov Identifier: NCT01569503). Nine patients were implanted with a VNS device and electrode (Cyberonics, Houston, TX, United States). Two patients were in failure of immunosuppressant (azathioprine) at the time of implantation and the other seven patients were naïve of treatment. Under general anesthesia, an electrode (Model 302) was wrapped around the left VN in the neck and connected to a bipolar pulse generator (Model 102) subcutaneously implanted in the chest wall. The day of the surgery, the device was switched on at 0.25 mA (duty cycle 30 s ON/5 min OFF, pulse width 250–500 μs -depending on patient tolerance-, 10 Hz frequency) and progressively increased up to 1.25 mA as patient tolerance permitted. VNS was continuously performed for 12 months. The first patient was implanted in April 2012 and the last in March 2016. Two patients were removed from the study after 3 months of neurostimulation for a worsening of their disease: the first patient underwent an ileo-cecal resection but, because of an initially beneficial effect and a drug treatment rejection, chose to continue neurostimulation until the end of the study. The second patient was treated with a combination of Infliximab and azathioprine and also wanted to keep on an active VNS. Six patients were in remission only under neurostimulation with a 1-year follow-up, the last patient was in flare. The first patient implanted in April 2012, was in relapse under azathioprine for an ileal CD with a history of ileo-cecal resection. We reported the results of this pilot study at 6-months follow-up in seven implanted patients (Bonaz et al., 2016a) and at 1 year in the all (*n* = 9) implanted (Sinniger et al., 2020). Briefly, of the seven patients who completed the 12-month VNS, five achieved clinical remission (CD activity index, CDAI < 150) and all the patients reached the CDAI-70 response (CDAI decrease from baseline by 70 points). Similarly the Crohn’s disease endoscopic score of severity (CDEIS) decreased in five patients from 60 to 100%. No adverse events related to the device were observed except discomfort to the intensity/output current levels. Our results are in accordance with the preliminary results of D’Haens study (D’Haens et al., 2018) who observed clinical and endoscopic improvement for half of the 16 CD patients under either VNS monotherapy (biologics refractory patients) or VNS adjunctive therapy for 4 months.

**FIGURE 5:**
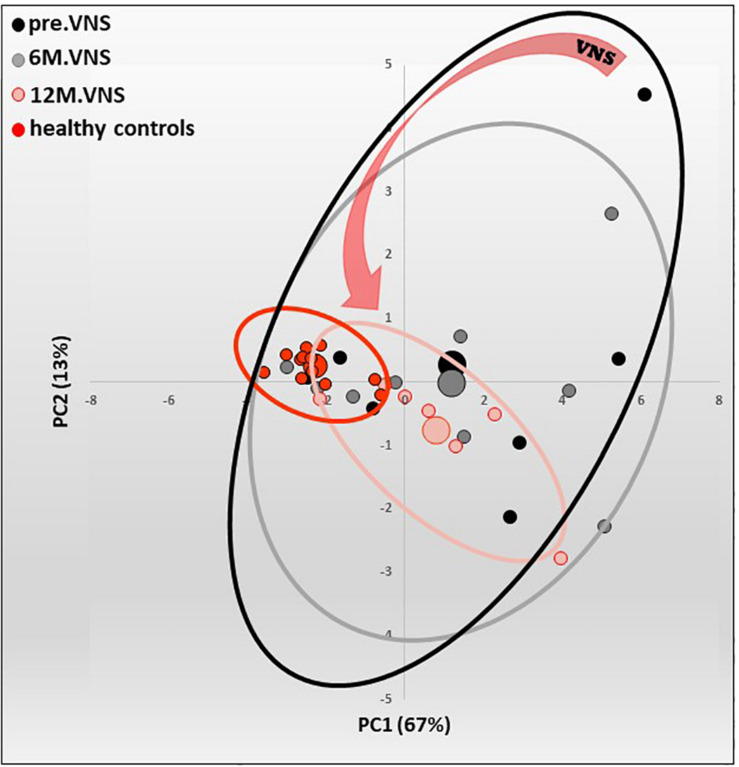
Pilot study of vagus nerve stimulation (VNS) in patients with moderate to severe Crohn’s Disease (CD). Twelve-month-VNS effect on cytokinergic profile. A plasma cytokinergic profile for controls (red), before (black), 6-month (gray) and 12-month (pink) VNS has been assessed using PCA analysis of plasma multicytokines assay for all CD patients [IL1b, IL2, IL6, IL10, IL12(p70), IL17A, IL21, IL23, MIP1α, IFNγ, GM-CSF, TNFα, TGFβ1 and MCP1]. The control values are well grouped, while profiles before VNS are very scattered, indicating that CD patients have their own cytokinergic profile. After 6 months, and even more after 12 months of VNS, the points are tightened, indicating that cytokine levels evolve through a more “common” profile. Ellipses are centered on the barycenter (big dots) of each group. Adapted from Sinniger et al. (2020).

A 12-month VNS could reduce inflammatory markers like CRP (in four patients whose three reaching normal value), fecal calprotectin (in three patients), and cytokines like TNF, IL6, IL12, and IL23, all being archetypal pro-inflammatory cytokines implicated in CD (Figure 5) (Sinniger et al., 2020). VNS was also able to increase or sustain plasma anti-inflammatory TGF-β1 in six patients, probably through its active regulatory role on Th17/Treg balance as demonstrated by Bettelli et al. (2006) and Tanaka and Kishimoto (2012) and also through its regulatory role in monocyte-driven inflammatory responses resulting in a reduction in TNF and a production of IL10 (Lee et al., 2013). Some plasma inflammatory markers have been correlated with gut mucosa metabolic markers; this is the case for CRP, which correlated with taurine, a metabolite produced by CRP-activated leucocytes and involved in the cytoprotection and homeostatic maintenance of cells during inflammatory/oxidative processes. We also showed a correlation between TNF and lactate, alanine and β-hydroxybutyrate that could reflect a metabolite shift occurring within the gut mucosa during the 12-months VNS, both being either involved in activation/deactivation and in the redox state of the immune cells, or as an alternative source of energy.

This pilot study requires of course confirmation in a larger randomized double-blinded control study and, overall, a long-lasting follow-up of the patients to confirm these promising results (Cheng et al., 2020).

#### The Question of the Regulatory Role of VNS

**FIGURE 6:**
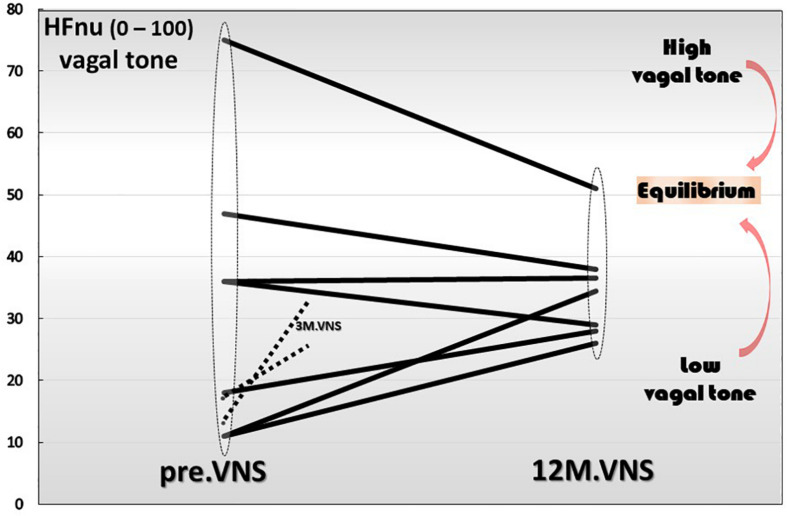
Twelve-month-vagus nerve stimulation (VNS) effect on vagal tone. High frequencies are expressed in normal units (HFnu) and are extracted from heart rate variability analysis.

Finally yet importantly, we have also shown that a 1-year chronic VNS exerts a modulatory role on vagal tone (Figure 6). Indeed, the trajectory toward the return to vagal equilibrium under VNS is dependent of the initial level of the HF-HRV. Interestingly, we observed in this clinical trial, that a very low HF-HRV on inclusion, increases until the equilibrium under VNS, a moderate level of HF-HRV was stabilized while an abnormally high resting HF-HRV on inclusion was decreased and brought back to equilibrium. Consequently, we can see that chronic VNS, on the long term, bring the autonomic regulation to homeostasis. At this stage, the question that arises is that of the mechanism by which this regulatory effect occurs, which has so far, never been observed before. A central mechanism through a change in the network balance within the CAN is most likely. There are several arguments in favor of this hypothesis. First, if we look at the kinetics of the evolution of the HRV over time, we can see that the return to equilibrium began at the third month of VNS (Sinniger et al., 2020). Second, we must also keep in mind that the assessment of HRV is mainly an evaluation of the ability of the central loops to regulate the functioning of the ANS, the HRV being the output (Figure 1). Hence, VNS logically must imply a mechanism that takes time to set up. VNS, used in the treatment of drug-refractory epilepsy, drives a 50% reduction frequency in 40–60% of the patients, with an increasing efficacy up to 10 years, showing that this treatment is a slow-acting therapy (Elliott et al., 2011). Third, electroencephalographic studies performed along with VNS over 1-year follow-up, revealed differences on power spectral bands between acute and chronic VNS. Acute VNS increased delta and theta bands on frontal, temporal and occipital sites while 1 year chronic VNS decreased power in the alpha band in correlation with the improvement of bowel mucosal inflammation, anxiety state and vagal tone. This suggests that chronic VNS has a regulatory action through the CNS and probably the CAN via afferent vagal fibers (Kibleur et al., 2018). This regulation-modulation mechanism of VNS on the return to equilibrium of the ANS but also that of the cytokines is quite original and rare in therapy outside of the example of thymoregulatory drugs like lithium. This requires serious consideration of this fundamental question by continuing investigations in this field.

## Conclusion

Targeting the anti-inflammatory properties of the VN with VNS could be of interest in the management of patients with CD through a non-drug therapy. VNS is an alternative to biologics such as anti-TNF but also other pro-inflammatory cytokines such as IL-6, IL-12, IL-23, as observed in our study, or even as an alternative to any drug treatment: this was the case in five of our first seven patients who were naïve of treatment on inclusion. In addition, the CAP is an intrinsic anti-inflammatory non-drug pathway, which protects against the potential iatrogenic effects of treatments. VNS, on the other hand, is devoid of major side effects and cheaper than biologics (the electrode and neurostimulator cost ∼ 11,000 euros).

Non-invasive neurostimulation by the transcutaneous auricular route (ta-VNS) is an alternative to invasive neurostimulation, as used in our pilot study (Butt et al., 2020). The aim of ta-VNS is to stimulate the ear *concha* (*concha auriculae*), part of the ear which is 100% innervated by the auricular branch of the VN (Peuker and Filler, 2002) whose stimulation would activate the “inflammatory reflex.” The tragus and the cavity of the concha are 45% innervated by the auricular branch of the VN. A recent functional brain imaging study showed that neurostimulation of this region of the ear induced brain activation of the NTS and its numerous projection sites, as observed with invasive VNS (Badran et al., 2018). Ta-VNS is under clinical investigation in a double blind placebo-controlled study in adult patients with UC (ClinicalTrials.gov Identifier: NCT03908073) and pediatric patients with IBD (CD and UC) (ClinicalTrials.gov Identifier: NCT03863704). It is also possible to stimulate the VN at the left cervical level with the Gammacore device marketed by Electrocore LLC (Basking Ridge, NJ, United States) represented by two round stainless steel disks serving as a contact surface with the skin. This device, recommended in the treatment of headaches, epilepsy, and depression (Ben-Menachem et al., 2015), delivers a stimulation lasting 2 min with a frequency of 20 Hz. There is presently no clinical trial registered with this technique in Clinical.Trial.gov.

The optimal parameters of VNS to achieve efficacious inflammation-related symptomatic relief by recruiting the appropriate fibers within the VN are still unknown. Specific combinations of pulse width, pulse amplitude, and frequency produced significant increases of the proinflammatory cytokine TNF, while other parameters selectively lowered serum TNF levels, as compared to sham-stimulated mice (Bonaz, 2020; Tsaava et al., 2020). VN morphology influences fiber responses to electrical stimulation. Specifically, nerve diameter (and thus, electrode-fiber distance), fascicle diameter, fascicular organization, and perineurium thickness all significantly affect the responses of nerve fibers to electrical signals delivered through a cuff electrode (Pelot et al., 2020). Miniaturization of the VNS device is also warranted. In the same way, instead of an electrode, a VNS device which would act as an electrode by clipping it around the VN would be of interest (see setpointmedical.com; MØ1-ØØ1123). Another important progress would be a device able to record HRV and trigger VNS in case of low HRV to restore a normal tone. A VNS system, AspireSRTM, already approved in Europe, and created by Cyberonics Inc. analyzes relative changes of heart rate, particularly ictal tachycardia, and responds to seizures automatically. Consequently, all the technical and anatomical points developed above should be taken into consideration in future clinical studies and may influence the results of these studies.

## Author Contributions

BB wrote the first draft of the manuscript. VS and SP completed the writing of the manuscript and built the figures. All authors contributed to the article and approved the submitted version.

## Conflict of Interest

The authors declare that the research was conducted in the absence of any commercial or financial relationships that could be construed as a potential conflict of interest.

## Abbreviations

ACh: acetylcholine
α 7nAChR: alpha7 nicotinic ACh receptor
ACTH: adrenocorticotropic hormone
ANS: autonomic nervous system
CAN: central autonomic network
CAP: cholinergic anti-inflammatory pathway
CD: Crohn’s disease
CNS: central nervous system
CRF: corticotrophin-releasing factor
CRP: C-reactive protein
DMNV: dorsal motor nucleus of the vagus
DSS: dextran sulfate sodium
HPA: hypothalamic pituitary adrenal
HRV: heart rate variability
IL: interleukin
IBD: inflammatory bowel diseases
LC: locus coeruleus
LPS: lipopolysaccharides
MDD: major depressive disorder
NE: norepinephrine
NTS: nucleus of the solitary tract
PVH: paraventricular nucleus of the hypothalamus
ta-VNS: transcutaneous auricular vagus nerve stimulation
TNBS: trinitrobenzensulfonic
TNF: tumor necrosis factor
UC: ulcerative colitis
VN: vagus nerve
VNS: vagus nerve stimulation.
